# The impact of the introduction of new recognition criteria for overwork-related cardiovascular and cerebrovascular diseases: a cross-country comparison

**DOI:** 10.1038/s41598-017-00198-5

**Published:** 2017-03-13

**Authors:** Ro-Ting Lin, Cheng-Kuan Lin, David C. Christiani, Ichiro Kawachi, Yawen Cheng, Stéphane Verguet, Simcha Jong

**Affiliations:** 1 Takemi Program in International Health, Department of Global Health and Population, Harvard T.H. Chan School of Public Health, 665 Huntington Avenue, Building 1, Room 1210A, Boston, Massachusetts 02115 USA; 2 National Institute of Environmental Health Sciences, National Health Research Institutes, 35 Keyan Road, Zhunan, Miaoli County 35053 Taiwan; 3 Department of Occupational Safety and Health, China Medical University, No. 91 Hsueh-Shih Road, Taichung, 40402 Taiwan; 4 Department of Environmental Health, Harvard T.H. Chan School of Public Health, 665 Huntington Avenue, Building 1, Room 1401, Boston, Massachusetts 02115 USA; 5 Department of Epidemiology, Harvard T.H. Chan School of Public Health, 665 Huntington Avenue, Building 1, Room 1401, Boston, Massachusetts 02115 USA; 6 Department of Social and Behavioral Sciences, Harvard T.H. Chan School of Public Health, Boston, 677 Huntington Avenue, Kresge Building, 7th Floor, Boston, Massachusetts 02115 USA; 7 Institute of Health Policy and Management, College of Public Health, National Taiwan University, Room 617, No. 17, Xuzhou Road, Taipei, 10055 Taiwan; 8 Department of Global Health and Population, Harvard T.H. Chan School of Public Health, 665 Huntington Avenue, Building 1, Room 1206D, Boston, Massachusetts 02115 USA; 9 Department of Global Health and Population, Harvard T.H. Chan School of Public Health, 665 Huntington Avenue, Building 1, Room 1215, Boston, Massachusetts 02115 USA; 10 Science Based Business, Leiden University, Snellius Building, Niels Bohrweg 1, 2333 CA, Leiden Netherlands

## Abstract

Cardiovascular and cerebrovascular diseases (CVDs) related to overwork are common in Asia, as is death from overwork, known as *karoshi*. Japan was the first country in the world to introduce criteria for recognizing overwork-related CVDs in 1961. Taiwan followed Japan in putting in place new policies and then updating these in 2010. We aimed to investigate the effect of introducing the new criteria for recognizing overwork-related CVDs in both countries. We defined the baseline period as the 5 years before launch of the new criteria, then collected data to 5 years after the new criteria. We applied a Poisson regression model to analyze the longitudinal change in rates of overwork-related CVDs before and after, adjusting for indicators of working conditions. Implementation of the new criteria was associated with a 2.58-fold increase in the rate of overwork-related CVDs (*p*-value < 0.05). However, the examined policy framework in Taiwan still appears to miss a substantial number of cases compared to that are captured by a similar policy framework used to capture overwork-related CVD rates in Japan by a factor of 0.42 (*p*-value < 0.05). Accordingly, we make a case for enhancements of Taiwan’s system for reporting and recognizing overwork-related diseases and deaths.

## Introduction

Cardiovascular and cerebrovascular diseases (CVDs) are the leading cause of death in the world. An estimated 17.3 million people died from CVDs in 2013, accounting for one third of total global deaths^1^. CVDs are associated with metabolic, behavioral, environmental, and occupational risk factors^2^. Among the occupational risk factors, long working hours can expose workers to workplace stress and also cause sleep deprivation, which may result in increased risk of depression, hypertension, and heart disease^3^. A recent meta-analysis of European, US, and Australian studies reported a strong dose-response association between people working long hours and risk of CVDs^4^. This analysis found that as working hours exceeded 35 to 40 hours per week, CVD risks increased: by 10% for 41–48 hours per week, 27% for 49–54 hours, and 33% for 55 hours and above^4^. Long-term CVD risk has been found to increase beginning at 46 working hours per week among employees working for 10 years or longer^5^.

Given that longer working hours appear to increase people’s risk of CVDs, national policies regarding sensible working hours are increasingly seen as important to protecting workers’ health. Many countries have long since made the 40-hour week the law^6^. Taiwan’s long work week lags in this regard. Compared to their counterparts in Organization for Economic Co-operation and Development (OECD) countries in Asia, Taiwanese workers spend the greatest amount of time in the workplace: an average of 2,135 hours annually in 2014 (or the equivalent of 41 hours of work each week for 52 consecutive weeks)^7,8^. This number was 365 hours (or the equivalent of 46 working days) more than the OECD average for that year.

Taiwan, Japan, and Korea are the only three countries in the world in which national governments have officially announced criteria for recognizing and compensating CVDs caused by overwork^9^. The criteria adopted by Taiwan for recognizing overwork-related CVDs were similar to the criteria adopted in Japan; on the other hand, Korea adopted different criteria^9^. In addition, Taiwan and Japan shared the similar recognizing process, e.g., the application of occupational disease compensation was reviewed by the Ministry of Labor in Taiwan. Only for the disputable case, the application was further examined by a committee for the central government that usually involved in physicians specialized in occupational diseases. An understanding of whether the gap exists between countries in applying the same criteria could help the government capture the effectiveness of the recognition system for overwork-related CVDs. Whereas previously, a CVD was only recognized as related to overwork when onset followed 24 consecutive hours of work, in 2004, Taiwan adopted Japan’s use of the number of long-term accumulated working hours as a key criterion for recognizing a CVD as related to overwork. Specifically, a CVD was recognized as such (Table 1) if an employee worked 100 hours or more of overtime during the month prior to the onset of CVD, or worked an average of 45 overtime hours or more per month during the 6 months preceding the onset of CVD^9^. However, Taiwan’s maximum working hours per month, excluding overtime, remained 8 hours higher than in Japan until 2016 when these were brought in line. Thus, over the studied period the overall threshold for recognizing a CVD case as linked to overwork was higher in Taiwan than in Japan.

**Table 1:** Comparison of major changes in recognition criteria for overwork-related cardiovascular and cerebrovascular and cerebrovascular diseases (CVDs) in Taiwan.

Item	Amendment of 2004	Amendment of 2010
Date of Amendment	31 December 2004	17 December 2010
Name of CVDs attributable to overwork*	Cardiovascular diseases	Cardiovascular diseases
	1. Sudden cardiac death	1. Myocardial infarction
	2. Acute myocardial infarction	2. Acute heart failure
	3. Acute heart failure	3. Dissecting aneurysm of the aorta
	4. Dissecting aneurysm of the aorta	4. Angina pectoris
		5. Cardiac arrest
		6. Sudden cardiac death
		7. Serious cardiac arrhythmia
	Cerebrovascular diseases	Cerebrovascular diseases
	1. Cerebral hemorrhage	1. Cerebral hemorrhage
	2. Cerebral thrombosis	2. Cerebral infarction
	3. Cerebral embolism	3. Subarachnoid hemorrhage
	4. Subarachnoid hemorrhage	4. Brain damage caused by severe hypertension
	5. Cerebral infarction	
	6. Brain damage caused by severe hypertension	
		Diseases not mentioned above in which work-related factors contributed to 50% or more of the development of the disease will also considered to be work-related.
Overwork criteria: Working hours	Maximum working hours: 84 hours every two weeks**.	Maximum working hours: 84 hours every two weeks.**
	Overtime:	Overtime:
	1. Overtime working hours reached 100 hours or more during the 1 month prior to the event.	1. Overtime working hours reached 92 hours or more during the 1 month prior to the event.
	2. Overtime working hours reached an average of 80 hours or more per month during the 2^nd^ to 6^th^ months prior to the event.	2. Overtime working hours reached an average of 72 hours or more per month during the 2^nd^ to 6^th^ months prior to the event.
	3. Overtime working hours reached an average of 45 hours or more per month during past 6^th^ months prior to the event.	3. Overtime working hours reached an average of 37 hours or more per month during past 6^th^ months prior to the event.
Overwork criteria: Psychological factors	No assessment tool	Provided an assessment tool and categorized the risk into following three types:
		1. An unusual event: an event that triggers extreme psychological tensions, excitement, fear, stress, the taking on of an extreme physical workload, or a dramatic change in work environment.
		2. Short-term overwork: extreme overtime work prior to CVD onset, or situations that cause mental or psychological overload based on assessment tool.
		3. Long -term overwork: overtime work prior to the CVD onset (see overtime criteria), or situations that cause mental or psychological overload based on assessment tool.
Area of onset	Not defined	Not limited to the workplace
Burden of proof	Not defined	Employer

^*^A disease in which more than 50% of the risk of development of the disease is attributable to non-work-related factors will not be considered a work-related disease.

**On January 1, 2016, the Labor Standards Act was amended to cut the former maximum work limit of 84 hours every two weeks to 40 hours per week.

As a result of pressure from the public, the Ministry of Labor of Taiwan amended its criteria for recognizing overwork-related CVD cases again in December 2010 (Table 1) to account for the total amount of hours worked, and not overtime alone^10^. The new criteria were meant to make it easier for occupational physicians and industrial hygienists to evaluate the association between overwork and onset of a CVD, and for workers to be compensated more easily. Between 2006 and 2015, Taiwan recognized 551 overwork-related CVD cases^11^. Taiwan still has relatively fewer cases of recognized overwork-related CVDs than Japan, for two reasons: First, the average hours worked per month, including overtime work, is 20 hours more in Taiwan than in Japan^7,12^. Second, the rate of all compensated occupational diseases compared to total insured workers in Taiwan (5.88 per 100,000 in 2010) was only 41% of the equivalent rate in Japan (14.19 per 100,000 in 2009)^13^. Although previous studies have identified the problem of substantial underestimation of overwork CVDs in Taiwan, the gap between Taiwan and Japan remained unexplored^9,13^.

Data on the development of overwork-related CVD rates in Japan and Taiwan provide us an opportunity to examine the impact of changes in criteria for recognizing overwork-related CVDs on recognized CVD rates linked to overwork in general, and the impact of the outlined 2010 policy change in Taiwan in particular, which has not previously been investigated. To this aim, we compared Taiwanese rates of overwork-related CVDs before and after the policy change as well against the rates in Japan before and after the similar policy change. We then used these data to assess whether overwork-related CVDs were potentially undercounted in Taiwan and by how much, taking into account data on working hours, salary, and unemployment rates in both countries.

## Methods

### Data: study period and subject units

We used an ecological study design to compare the change in recognized overwork-related CVDs from before to after the implementation of the new criteria, adjusting for work-related confounding factors. We defined the baseline period as the 5-year period prior to launch of the new criteria—new criteria were implemented in December 2010 in Taiwan and in December 2001 in Japan. Data were collected for 5 years after introduction of the new criteria. The study periods, then, were from 2006 through 2015 for Taiwan and from 1997 through 2006 for Japan.

The unit of analysis is at the level of industry sector. We followed each country’s Standard Industry Codes to group industries into six sectors: (1) manufacturing; (2) construction; (3) transportation and storage, information and communication, and art; (4) wholesale and retail trade and accommodation and food service activities; (5) financial and insurance activities and human health and social work activities; and (6) other (see Table 2)^14^. We excluded four industries from our analyses due to lack of complete data: agriculture, forestry, and fishing; mining and quarrying; public administration and defense/compulsory social security; and education. The six sectors included in the analyses covered 88% of 2015 employees in Taiwan and 94% of 2006 employees in Japan.

**Table 2:** Industries included in the analysis, by industry sector.

Name of sector	Industries in sector*
Manufacturing	Manufacturing
Construction	Construction
Transportation, information, arts	Transportation and storage
	Information and communication
	Arts, entertainment, and recreation
Trade, accommodation, food	Wholesale and retail trade; repair of motor vehicles and motorcycles
	Accommodation and food service activities
Financial, insurance, health	Financial and insurance activities
	Human health and social work activities
Other	Electricity, gas, steam, and air conditioning supply
	Water supply; sewerage, waste management, and remediation activities
	Real estate activities
	Professional, scientific, and technical activities
	Administrative and support service activities
	Other service activities

*Based on the United Nations’ international standard industrial classification of all economic activities, rev. 4^14^. Industries lacking complete data were excluded from our analysis. These included: agriculture, forestry, and fishing; mining and quarrying; public administration and defense/compulsory social security; and education.

### Data: dependent variables

In this study, we defined the number of cases of overwork-related CVDs as the sum of person-cases of illness, disability, or death from CVDs. (Table 1 lists the diseases included in the Taiwan criteria.) We collected the number of overwork-related CVD cases and the number of hired employees for each industry sector from the Ministry of Labor of Taiwan and the Ministry of Health, Labour, and Welfare of Japan, respectively^7,11,12,15^. We further calculated the rate of overwork-related CVDs by adding up all the overwork-related CVD cases in each year by industry sector and then dividing these numbers by the total number of hired employees for each sector.

We also collected the number of overall occupational diseases recognized in Taiwan. By comparing the changes in the rate of overwork-related CVDs to the rate of occupational diseases overall, we could assess whether any changes we found could be attributed to overall, versus CVD-specific, improvements in occupational disease reporting.

### Data: independent variables

Our primary exposure measure was the implementation of new criteria for designating CVDs as overwork-related. (Table 1 describes the differences between the old and new criteria adopted in Taiwan.) According to the criteria, working hours and psychological factors were risk factors for developing overwork-related CVDs. As working hours was a directly measurable indicator and the data were available for each industry sector in both countries, we identified two ecological variables of working conditions as proxy indicators of the relative state of psychological factors; these were salary and unemployment rate. Employment provided an opportunity to access financial and social sources that could further benefit health^16^; and, the salary represented financial functions of the work^17^. Consumer Price Index (CPI) was the most commonly used measure of price change over time. We used the CPI-adjusted salary to represent the microenvironment change of the economy in each country^18–21^. We collected these data for each industry sector in each country from the Ministry of Labor of Taiwan and the Ministry of Health, Labour, and Welfare of Japan^7,12^.

### Data analysis

We averaged the numbers and rates of overwork-related CVDs for each industry sector both before and after the implementation of the new criteria. The crude rate ratio was derived from the rate after implementation divided by the rate before implementation, while the rate difference was derived from the subtraction of the rate before implementation from the rate after implementation. We used Wilcoxon signed-rank tests to examine the differences.

We further used a generalized mixed-effects model with a Poisson distribution and longitudinal data to estimate the change in rates of overwork-related CVDs before and after implementing the new criteria, stratifying by different countries and taking into account selected covariates:

$$\begin{array}{rcl}{\rm{ln}}(E[{\lambda }_{ij}| {X}_{ij}]) & = & {\rm{ln}}(worke{r}_{ij})+{\beta }_{0}+{b}_{0i}+{\beta }_{1}\,new\,criteri{a}_{i}+{\beta }_{2}\,working\,hou{r}_{ij}\\  &  & +\,{\beta }_{3}\,salar{y}_{ij}+{\beta }_{4}\,unemployment\,rat{e}_{ij}+{\beta }_{5}\,yea{r}_{i}\end{array}$$

In this equation, *i* denotes the code of industry sector in each country; *j* denotes time in years since the baseline year of the 10-year study period (from 0 to 9); *E*[*Y*_*ij*_*|X*_*ij*_] denotes the expected overwork-related CVD cases conditioned on covariates *X*; ln(*worker*_*ij*_), as offset, denotes the natural logarithm of all employed workers in industry sector *i* and in time *j*; *β*_*0*_ is the intercept for the fixed effect; *b*_*0i*_ is the random intercept of industry sectors in each country; and *β*_*1*_ to *β*_*5*_ are estimated coefficients for the marginal effects on the population derived from maximum likelihood method. The incorporation of random intercept in our model was to control the heterogeneity of industry sectors. After establishing country-specific model, we repeated the analysis for data on two countries combined and added one covariate of “country recognition system” as a surrogate of the systemic error within a country. We performed the PROC GLIMMIX procedure using SAS version 9.4 (SAS Institute, Cary, NC, US) to estimate the effect of selected factors on rates of overwork-related CVDs. For the standard errors and resultant 95% confidence intervals, we used an unstructured variance covariance matrix.

### Ethical approval

This study was deemed exempt by the Institutional Review Board (IRB) of the Harvard T.H. Chan School of Public Health, USA (No. IRB16–0430), per the regulations found at 45 CFR 46.101(b)(4).

## Results

Table 3 shows characteristics of the workforce and working conditions by industry sector in Taiwan and Japan, both before and after the implementation of the new criteria. Most of the employees in both countries were in manufacturing; in trade, accommodation, and food; or in the “other” sector—these employees together accounted for more than 70% of the workforce.

**Table 3:** Characteristics of the workforce and working conditions in Taiwan and Japan before and after implementation of new criteria for overwork-related cardiovascular and cerebrovascular diseases, by industry sector.

Characteristics	Taiwan				Japan			
	Before*		After^†^		Before*		After^†^	
Number of employees	Mean (thousand)	%	Mean (thousand)	%	Mean (thousand)	%	Mean (thousand)	%
**Total**	**6828.20**	**100.00**	**7529.80**	**100.00**	**42551.00**	**100.00**	**42004.80**	**100.00**
Manufacturing	2553.60	37.40	2737.80	36.36	10259.00	24.11	8920.00	21.24
Construction	686.60	10.06	726.80	9.65	4124.60	9.69	3364.00	8.01
Transportation, information, arts	572.60	8.39	632.80	8.40	3473.40	8.16	3686.40	8.78
Trade, accommodation, food	1377.40	20.17	1580.60	20.99	9999.60	23.50	11334.20	26.98
Financial, insurance, health	730.40	10.70	812.60	10.79	1643.40	3.86	3179.00	7.57
Other	907.60	13.29	1039.20	13.80	13051.00	30.67	11521.20	27.43
**Working hours per month**	**Mean (hour)**	**SD** ^**‡**^	**Mean (hour)**	**SD** ^**‡**^	**Mean (hour)**	**SD** ^**‡**^	**Mean (hour)**	**SD** ^**‡**^
**Total**	**178.19**	**8.58**	**176.71**	**9.71**	**157.47**	**8.79**	**156.41**	**12.22**
Manufacturing	185.50	3.90	183.90	1.29	162.92	1.46	165.02	1.62
Construction	176.68	1.17	175.86	2.47	169.04	1.19	170.72	1.13
Transportation, information, arts	177.50	0.52	175.20	1.33	169.60	0.63	171.52	1.56
Trade, accommodation, food	173.96	0.81	171.56	1.20	143.48	3.67	134.99	1.44
Financial, insurance, health	171.30	0.46	169.12	1.36	150.32	0.68	147.61	2.84
Other	184.18	1.72	184.59	2.06	149.46	1.16	148.58	1.73
**Salary per month**	**Mean (US dollar)** ^**§**^	**SD** ^**‡**^	**Mean (US dollar)** ^**§**^	**SD** ^**‡**^	**Mean (US dollar)** ^**§**^	**SD** ^**‡**^	**Mean (US dollar)** ^**§**^	**SD** ^**‡**^
**Total**	**1477.44**	**319.25**	**1588.63**	**353.10**	**3139.83**	**556.43**	**3091.98**	**560.99**
Manufacturing	1346.51	76.50	1439.44	20.65	3039.30	224.90	3252.47	247.61
Construction	1298.01	25.66	1387.10	51.84	3104.08	250.46	3102.06	206.01
Transportation, information, arts	1551.56	73.29	1748.34	65.75	3258.13	222.09	3293.11	243.74
Trade, accommodation, food	1084.05	71.34	1279.22	24.61	2295.26	133.72	2107.68	82.26
Financial, insurance, health	2068.09	77.19	2289.07	34.83	4035.78	301.65	3784.40	537.26
Other	1516.39	126.64	1388.57	35.10	3106.41	212.29	3012.12	169.67
**Unemployment rate**	**Mean (%)**	**SD** ^**‡**^	**Mean (%)**	**SD** ^**‡**^	**Mean (%)**	**SD** ^**‡**^	**Mean (%)**	**SD** ^**‡**^
**Total**	**4.13**	**1.58**	**3.49**	**0.83**	**2.06**	**0.35**	**2.08**	**0.41**
Manufacturing	4.14	1.03	3.25	0.24	1.49	0.05	1.43	0.11
Construction	6.27	2.40	4.82	0.54	2.15	0.09	1.94	0.20
Transportation, information, arts	4.18	0.76	3.46	0.25	1.89	0.09	1.82	0.07
Trade, accommodation, food	3.90	0.64	3.57	0.21	2.62	0.11	2.89	0.06
Financial, insurance, health	2.49	0.44	2.21	0.35	2.16	0.08	2.13	0.16
Other	3.81	0.89	3.64	0.23	2.05	0.06	2.26	0.09

*Defined as 5 years before the new criteria: 2006–2010 for Taiwan and 1997–2001 for Japan. ^†^Defined as 5 years after new criteria: 2011–2015 for Taiwan and 2002–2006 for Japan. ^‡^SD = standard deviation. ^§^Based on average annual exchange rate and adjusted for Consumer Price Index^18–21^.

In terms of working conditions, Taiwan had longer working hours and higher unemployment rates than Japan. Even though average working hours slightly decreased across most industry sectors in Taiwan after the implementation of the new criteria, average working hours in Taiwan were still about 20 hours more per month than in Japan. This difference between countries was more apparent in the trade, accommodation, and food sector (36.57 hours more in Taiwan) and in the “other” sector (36.01 hours more in Taiwan) than in the other sector groups. Average salary increased in Taiwan in the period after the new criteria were implemented; in contrast, the average salary in Japan slightly decreased after its new criteria were implemented. The unemployment rate in Taiwan decreased after implementing the new criteria, but was still 1.41 percentage points higher than in Japan.

Table 4 shows the average numbers and rates of recognized overwork-related CVDs in Taiwan before and after the new criteria, compared to numbers and rates of other occupational diseases in Taiwan and to overwork-related CVDs in Japan. After applying the new criteria, the rate of overwork-related CVDs in Taiwan had a 2.63-fold increase (rate ratio = 2.63, *p*-value < 0.05), with an additional 7.12 overwork-related CVD cases per million total employees in Taiwan (rate difference = 7.12). During the same period, other occupational diseases in Taiwan had a 1.77-fold increase (rate ratio = 1.77, *p*-value < 0.05), while in the years following implementation of Japan’s new criteria, the rate of overwork-related CVDs in Japan had a 3.65-fold increase (rate ratio = 3.65, *p*-value < 0.05).

**Table 4:** Numbers, rates, rate ratios, and rate differences for overwork-related cardiovascular and cerebrovascular diseases (CVDs) and other occupational diseases in Taiwan and Japan before and after implementation of new criteria for overwork-related CVDs.

	Before*				After^†^				Rate ratio	Rate difference	*p*-value^§^
	Number per year		Rate per year^‡^		Number per year		Rate per year^‡^				
	Mean	SD	Mean	SD	Mean	SD	Mean	SD			
**Taiwan: overwork-related CVDs**											
**Total**	**27.60**	**11.06**	**4.36**	**3.97**	**77.80**	**12.17**	**11.48**	**8.09**	**2.63**	**7.12**	**<0.05**
Manufacturing	7.80	2.39	3.04	0.89	22.80	7.33	8.34	2.74	2.74	5.30	<0.05
Construction	1.20	1.30	1.75	1.93	6.20	2.77	8.54	3.81	4.89	6.79	<0.05
Transportation, information, arts	5.60	3.21	9.64	5.42	12.00	6.63	19.36	11.50	2.01	9.72	0.13
Trade, accommodation, food	7.20	3.96	5.15	2.68	11.40	1.34	7.24	1.06	1.40	2.09	0.15
Financial, insurance, health	0.60	0.55	0.83	0.76	3.80	0.84	4.67	1.00	5.63	3.84	<0.05
Other	5.20	2.49	5.76	2.88	21.60	6.31	20.72	5.55	3.60	14.96	<0.05
**Taiwan: other occupational diseases**											
**Total**	**753.80**	**70.32**	**60.79**	**33.99**	**400.60**	**136.13**	**107.80**	**62.07**	**1.77**	**47.01**	<**0.05**
Manufacturing	151.20	43.49	59.11	16.56	252.20	33.92	92.05	11.54	1.56	32.94	<0.05
Construction	75.00	32.37	110.36	50.54	166.40	22.12	229.07	30.26	2.08	118.70	<0.05
Transportation, information, arts	34.80	12.78	60.50	21.08	60.00	18.61	95.11	29.97	1.57	34.61	0.10
Trade, accommodation, food	64.60	21.48	46.51	13.75	131.00	17.79	83.28	14.15	1.79	36.77	<0.05
Financial, insurance, health	25.20	12.52	34.14	15.77	31.40	2.51	38.65	3.15	1.13	4.51	0.42
Other	49.80	20.08	54.15	19.77	112.80	12.32	108.66	12.36	2.01	54.51	<0.05
**Japan: overwork-related CVDs**											
**Total**	**92.40**	**27.41**	**2.57**	**1.44**	**313.92**	**34.59**	**9.38**	**7.04**	**3.65**	**6.81**	**<0.05**
Manufacturing	21.00	8.49	2.07	0.90	46.85	13.44	6.06	1.17	2.93	4.00	<0.05
Construction	9.20	2.59	2.23	0.65	33.85	7.24	10.05	1.83	4.50	7.82	<0.05
Transportation, information, arts	15.60	7.02	4.50	2.05	97.00	11.99	23.76	2.31	5.28	19.26	<0.05
Trade, accommodation, food	12.00	6.82	1.20	0.65	73.54	13.41	6.44	0.95	5.38	5.24	<0.05
Financial, insurance, health	5.20	1.64	3.18	1.08	11.31	3.47	4.51	3.23	1.42	1.32	0.69
Other	29.40	4.10	2.25	0.25	51.38	11.01	5.47	2.38	2.43	3.22	<0.05

SD = standard deviation. *Defined as 5 years before new criteria: 2006–2010 for Taiwan and 1997–2001 for Japan. ^†^Defined as 5 years after new criteria: 2011–2015 for Taiwan and 2002–2006 for Japan. ^‡^Per million employees. ^§^Wilcoxon signed-rank tests.

The rate ratio shows that in Taiwan, the financial, insurance, and health sector had the highest increase in rate of cases (rate ratio = 5.63, *p*-value < 0.05); however, for the same sector, there was no significant change in the rate for other occupational diseases in Taiwan (rate ratio = 1.13, *p*-value = 0.42) or for overwork-related CVDs in Japan (rate ratio = 1.42, *p*-value = 0.69). The rate difference shows that after implementing the new criteria in Taiwan, the nation’s “other” sector had the largest increase in number of overwork-related CVDs rates, with an additional 14.96 CVD cases per million employees (rate difference = 14.96, *p*-value < 0.05).

Table 5 shows the modeling results for the selected determinants of overwork-related CVDs for each country. By examining the new criteria and adjusting for other covariates, we found that the effect of implementing the new criteria was significantly associated with 2.53-fold (*p*-value < 0.05) and 2.81-fold (*p*-value < 0.05) increases in the rate of overwork-related CVDs in Taiwan and Japan, respectively. The rate of overwork-related CVDs was positively associated with working hours per month (*p*-value < 0.05) in both countries, but its relation with salary and unemployment rates didn’t reach statistical significance (*p*-value = 0.09 to 0.91).

**Table 5:** Estimated marginal effects of determinants on rates of overwork-related cardiovascular and cerebrovascular diseases in Taiwan and Japan.

Determinants	Taiwan		Japan	
	Estimated Rate (95% CI)	*p*-value	Estimated Rate (95% CI)	*p*-value
Implementation of new criteria (after vs. before)	2.5279 (1.6147–3.9577)	0.0002	2.8061 (1.7822–4.4182)	<0.0001
Working hours per month	1.0674 (1.0085–1.1298)	0.0289	1.0470 (1.0164–1.0785)	0.0038
Salary per month*	1.0004 (0.9994–1.0014)	0.4609	1.0000 (0.9996–1.0004)	0.9069
Unemployment rate	1.0638 (0.9148–1.2370)	0.4258	1.9408 (0.9147–4.1179)	0.0904
Year	1.0271 (0.9496–1.1110)	0.5073	1.0573 (0.9803–1.1404)	0.1552

The estimate intercepts are 1.3931 × 10^−5^ (95% CI = 3.0317 × 10^−10^ to 6.4013 × 10^−1^, *p*-value = 0.0967) for Taiwan and 4.0529 × 10^−4^ (95% CI = 1.4069 × 10^−6^ to 1.1676 × 10^−1^, *p*-value = 0.0426) for Japan, respectively. *Based on average annual exchange rate and adjusted for Consumer Price Index^18–21^.

In order to estimate the potential effect of differences in the system for recognizing work-related diseases between the two countries, we further combined data of the two countries and repeated the analysis with an additional covariate: “country recognition system.” Table 6 shows that the effect of implementing the new criteria was significantly associated with 2.58-fold (*p*-value < 0.05) increases in the rate of overwork-related CVDs. We also found a significant effect for the “country recognition system” (*p*-value < 0.05). Given similar working conditions and the formal implementation of the new criteria of recognizing overwork-related CVDs were similar across the two countries, the recognized rate in Taiwan was just 0.42-fold of the rate in Japan. This indicated that the recognition system in Taiwan was not as strong as it in Japan.

**Table 6:** Estimated marginal effects of determinants on rates of overwork-related cardiovascular and cerebrovascular diseases.

Determinants	Estimated Rate (95% CI)	*p*-value
Implementation of new criteria (after vs. before)	2.5818 (2.1848–3.0511)	<0.0001
Country recognition system (Taiwan vs. Japan)	0.4245 (0.1945–0.9267)	0.0338
Working hours per month	1.0315 (1.0153–1.0480)	0.0002
Salary per month*	0.9998 (0.9996–1.0000)	0.0675
Unemployment rate	1.1620 (1.0298–1.3111)	0.0166
Year	1.0578 (1.0284–1.0880)	0.0002

The estimate intercepts are 2.1381 × 10^−5^ (95% CI = 1.9139 × 10^−6^ to 2.3886 × 10^−4^, *p*-value < 0.0001). *Based on average annual exchange rate and adjusted for Consumer Price Index^18–21^.

**Figure 1:**
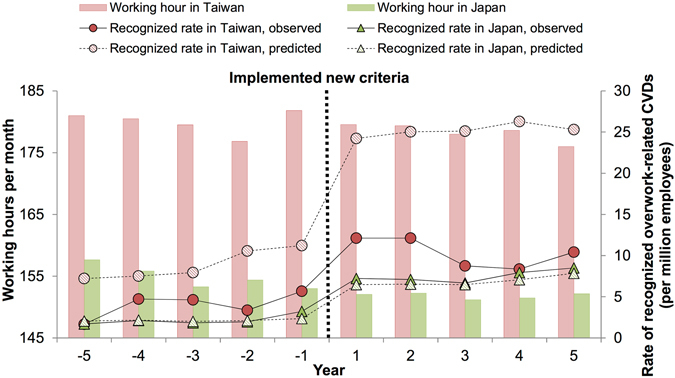
Trends of working hours per month and rates of recognized overwork-related cardiovascular and cerebrovascular diseases (CVDs) in Taiwan and Japan from the 5-year period before and after launch of the new criteria. Note: Predicted rates of recognized overwork-related CVDs (dashed lines) were based on the modeling results from Table 6, given that both countries were at the same level of recognition system for work-related diseases, i.e., Japanese system, and their working conditions remained unchanged.

Figure 1 shows the yearly time series for working hours per month (bars) and observed (solid lines) and predicted (dashed lines, based on the model in Table 6) rates of recognized overwork-related CVDs. While the average working hours in Taiwan were 20 or more hours per month more than in Japan, the rate of recognized overwork-related CVDs in both countries didn’t show an apparent difference. The gap between observed and predicted rates in Taiwan was more pronounced after the implementation of the new criteria. For example, if Taiwan reached the level of recognition system for work-related diseases of Japan and its working hours remained unchanged (i.e., 20 or more hours per months more than Japan), the predicted rates of overwork-related CVDs in Taiwan were 24.23–26.28 deaths per million employees per year, instead of the reported average of 11.48 deaths per million employees, for the periods 2011–2015.

## Discussion

This study found that the implementation of a policy with new criteria for recognizing overwork-related CVDs led to a 2.58-fold increase in the rate of overwork-related CVDs. New recognition criteria thus appear to help occupational physicians and industrial hygienists determine whether a CVD case is related to overwork or not, while also highlighting the problem of undercounting overwork-related CVDs under the old policy. Following both increasing awareness of occupational health and the establishment of Taiwan’s occupational health service network in 2007, the overall rate of recognized occupational diseases has increased^22^. Our study found a higher rate ratio for overwork-related CVDs than for overall occupational diseases (see Table 4), which further supports the conclusion that the new criteria for overwork-related CVDs had an additional effect on increasing the rate of recognized overwork-related CVDs. An increasing awareness of death from overwork among Taiwanese workers and their families might have also increased applications for recognizing CVD cases as overwork-related.

However, despite a doubling of the rate of overwork-related CVDs in Taiwan from 2010 to 2011, from 5.68 per million employees in 2010 to 12.12 per million employees in 2011, the actual rate of overwork-related CVDs might still be underreported. Since the criteria adopted by Taiwan for recognizing overwork-related CVD cases were similar to the criteria adopted in Japan in 2001, we expected to find a similar increase in recognition rates. Yet, after adjusting for all possible confounding factors, we found a substantial discrepancy between the two countries in their recognized rates of overwork-related CVD cases (Table 6, line 2). According to the approval rates for overwork-related CVDs, which was derived from the number of approved divided by the number of claims cases, the rates were about 38.57% in Japan during 2002–2006 but only 24.96% in Taiwan during 2012–2015^15,23^. The lower approval rate in Taiwan further supported our findings that overwork-related CVDs in Taiwan were undercounted. If Taiwan reached the level of recognition system for work-related diseases of Japan, we would have expected an increase in reported rates of overwork-related CVDs in Taiwan, to 25.19 per million (based on the average of predicted rate for the periods 2011–2015, see Fig. 1) after the implementation of the new criteria, based on the estimated risk ratio. In contrast, we found that the average for these rates was 11.48 per million (see Table 4), indicating that the problem of overwork-related CVDs in Taiwan remains under-recognized.

Researchers have described several factors that may contribute to under-recognition and underreporting of work-related diseases. These factors include insufficient records linking exposure to illness, lack of awareness, fear of retaliation or job loss, and the safety climate of an organization^24–26^. For example, Taiwan’s workers’ compensation system was integrated into the country’s national health care system in 1995. An employee with an occupational disease can now be diagnosed and treated under the national health services, and then can apply for compensation from the country’s labor insurance system. However, the application process is often tedious and prone to dispute by employers^27^. There are two other reasons workers often do not go through this process: First, most of the cost of health services can be covered by the national health services even if it is not recognized as work-related. Second, the additional compensation payment from the labor insurance system might be small. For these reasons, occupational diseases tend to be overlooked by the labor security and welfare system in Taiwan^28^.

After the new criteria were implemented in each country, Taiwanese employees still worked an average of 20 or more hours per month more than their Japanese counterparts (176.71 hours per month in Taiwan and 156.41 hours per month in Japan; see Table 3). Based on our analysis, employees who work 20 hours more have a 2.50-fold (=1.0470^20^, based on the parameter estimate of Japan in Table 5) greater risk of overwork-related CVDs. National surveys of working conditions in Taiwan have indicated that the distribution of working hours, between employees working short and long hours, became more polarized between 2001 and 2010^29^. The 2010 criteria for recognizing CVDs as overwork-related now serve as guidelines to help employers and policy makers regulate employees’ working hours in a way that incorporates the costs associated with overwork-related CVDs. Western European and North American countries with welfare systems have demonstrated that it is possible to achieve strong and stable labor regulation that balances working hours and work efficiency^30^.

The setting of this study—two countries that implemented a similar policy change—provided us with a unique window on the effect of policy changes that govern the reporting of work-related medical conditions. Specifically, our comparative data, and the problem we identified around underreporting of overwork-related CVDs in Taiwan, highlight critical challenges that policy makers face in designing and implementing initiatives that seek to improve the recognition of work-related conditions in national health care systems. For example, as shown in Table 4, although the old criteria adopted by Taiwan in 2004 followed the same overtime working hours under Japan’s revision in 2001, the rate of recognized overwork-related CVDs was only 4.36 during 2006–2010 in Taiwan, about only half of the rate in Japan (9.38 during 2002–2006). Such difference reflected differences in maximum limit of working hours and also actual working hours. Another example is that Taiwan had higher rates of recognized overwork-related CVDs than Japan before and after the implementation of the new criteria (Table 4). However, the recognized CVDs rates are affected by not only the new criteria but also the working hours. By teasing out the effect of country difference in maximum limit of working hours, excessive working hours, and recognition system, we were able to demonstrate a plausible evidence of recognition system of overwork-related CVDs in Taiwan underestimated systematically by a factor of 0.42. Excessive working hours are common in Asian countries as a side product of rapidly economic booming, especially in East Asia^31,32^. A cross-country collaboration and good benchmark on the establishment and implementation of the recognition system for overwork-related CVDs in line with working standards could help the policy makers to establish a more comprehensive and efficient system^31,32^.

Our ecological study design has some limitations. First, we analyzed industry-level data. Thus, our findings should not be extrapolated to the individual level or to industries not included in the analysis. Individual susceptibility to overwork and individual employees’ support systems might mediate the effect of the examined policy change on the reported numbers of occupational disease. The incorporation of personal data would allow for a more detailed interpretation of factors that cause variations among employees, and we recommend including such data in future qualitative studies that attempt to disentangle under-recognition or underreporting problems.

Second, country- and industry-level differences in labor structures and mortality rates might be associated with variations in number of recognized overwork-related CVDs. Although Japan has a higher proportion of older employees in the labor force than Taiwan does (see Supplementary Table S1), after the implementation of the new criteria in each country, both had similar rate ratios of overwork-related CVDs compared to overall CVDs (i.e., 0.8–0.9%; see Supplementary Table S2). In addition, under the new criteria, personal characteristics, such as age and sex, should have been evaluated by occupational physicians in the process of defining a CVD as overwork-related, to ensure that the causal role of occupational exposure was valid. This fact leads us to believe our results may be less susceptible to be biased.

Third, psychological factors, such as extreme psychological tension and stress in the work environment, might also play a role in the development of overwork-related CVDs^33,34^. According to a nationwide survey among Taiwanese workers, employees perceived higher levels of burnout if they worked 49 hours or more per week^35^. While this might have led us to overestimate the effect of working hours on overwork-related CVDs, we hope to have mitigated bias by taking into account working hours, salary, and unemployment rates as proxy indicators of the relative state of psychological factors.

## Conclusion

Our study shows that Taiwan’s new policy for recognizing overwork-related CVDs resulted in a 2.58-fold increase in the reported rates of overwork-related CVDs. Yet, our analyses also indicate a plausible under-recognition of overwork-related CVDs in Taiwan compared to Japan, by a factor of 0.42, under the new policy framework. Our demonstration of under-recognition and underreporting problems regarding overwork-related CVDs highlight the need for the government of Taiwan to enhance its recognition and reporting systems for occupational diseases. Moreover, our analyses underline the role of overwork in CVDs and the need for corporate and policy measures that address this cause of overwork-related CVDs.

## Electronic supplementary material

Supplementary Information
