# Author Correction: The impact of the introduction of new recognition criteria for overwork-related cardiovascular and cerebrovascular diseases: a cross-country comparison

**DOI:** 10.1038/s41598-018-23047-5

**Published:** 2018-03-12

**Authors:** Ro-Ting Lin, Cheng-Kuan Lin, David C. Christiani, Ichiro Kawachi, Yawen Cheng, Stéphane Verguet, Simcha Jong

**Affiliations:** 1 Takemi Program in International Health, Department of Global Health and Population, Harvard T.H. Chan School of Public Health, 665 Huntington Avenue, Building 1, Room 1210A, Boston, Massachusetts 02115 USA; 2 National Institute of Environmental Health Sciences, National Health Research Institutes, 35 Keyan Road, Zhunan, Miaoli County 35053 Taiwan; 3 Department of Occupational Safety and Health, China Medical University, No. 91 Hsueh-Shih Road, Taichung, 40402 Taiwan; 4 Department of Environmental Health, Harvard T.H. Chan School of Public Health, 665 Huntington Avenue, Building 1, Room 1401, Boston, Massachusetts 02115 USA; 5 Department of Epidemiology, Harvard T.H. Chan School of Public Health, 665 Huntington Avenue, Building 1, Room 1401, Boston, Massachusetts 02115 USA; 6 Department of Social and Behavioral Sciences, Harvard T.H. Chan School of Public Health, 677 Huntington Avenue, Kresge Building, 7th Floor, Boston, Massachusetts 02115 USA; 7 Institute of Health Policy and Management, College of Public Health, National Taiwan University, Room 617, No. 17, Xuzhou Road, Taipei, 10055 Taiwan; 8 Department of Global Health and Population, Harvard T.H. Chan School of Public Health, 665 Huntington Avenue, Building 1, Room 1206D, Boston, Massachusetts 02115 USA; 9 Department of Global Health and Population, Harvard T.H. Chan School of Public Health, 665 Huntington Avenue, Building 1, Room 1215, Boston, Massachusetts 02115 USA; 10 Science Based Business, Leiden University, Snellius Building, Niels Bohrweg 1, 2333 CA Leiden, Netherlands

Correction to: *Scientific Reports* 10.1038/s41598-017-00198-5, published online 13 March 2017

This Article contains an error in the Methods section under subheading ‘Data analysis’.

“ln(*E*[*λ*_*ij*_|*X*_*ij*_]) = ln(*worker*_*ij*_) + *β*_0_ + *b*_0*i*_ + *β*_1_ *new criteria*_*i*_ + *β*_2_ *working hour*_*ij*_ + *β*_3_ *salary*_*ij*_ + *β*_4_ *unemployment rate*_*ij*_ + *β*_5_ *year*_*i*_”

should read:

“ln(*E*[*Y*_*ij*_|*X*_*ij*_]) = ln(*worker*_*ij*_) + *β*_0_ + *b*_*0i*_ + *β*_1_ *new criteria*_*i*_ + *β*_2_ *working hour*_*ij*_ + *β*_3_ *salary*_*ij*_ + *β*_4_ *unemployment rate*_*ij*_ + *β*_5_ *year*_*i*_”

