# Supplementary material for: The impact of the introduction of new recognition criteria for overwork-related cardiovascular and cerebrovascular diseases: a cross-country comparison
Source: Sci Rep. 2017 Mar 13;7:167. doi: 10.1038/s41598-017-00198-5 (PMC5427845; doi:10.1038/s41598-017-00198-5)
Supplement: Supplementary file 1 — Supplementary Information [file 41598_2017_198_MOESM1_ESM.pdf]

Supplementary Information

Title

The impact of the introduction of new recognition criteria for overwork-related cardiovascular and cerebrovascular diseases: a cross-country comparison

Authors

Ro-Ting Lin*, Cheng-Kuan Lin, David C. Christiani, Ichiro Kawachi, Yawen Cheng, Stéphane Verguet, Simcha Jong

Corresponding author:

Ro-Ting Lin

Takemi Fellow, Department of Global Health and Population, Harvard T.H. Chan School of Public Health

Postdoctoral Research Fellow, National Health Research Institutes, Taiwan

Assistant Professor, Department of Occupational Safety and Health, China Medical University, Taiwan

Email: roting@ntu.edu.tw

Telephone: +886-4-22053366 ext. 6215

Table S1. Age structure of the workforce in Taiwan and Japan before and after major changes in recognition criteria for overwork-related cardiovascular and cerebrovascular diseases (CVDs)

Characteristics	Taiwan				Japan			
	Before*		After†		Before*		After†	
**Number of male employees, by age group**	**Mean (thousand)**	**%**	**Mean (thousand)**	**%**	**Mean (thousand)**	**%**	**Mean (thousand)**	**%**
25–34 years	1596.60	29.12	1564.00	27.22	10332.00	24.86	10175.40	24.75
35–44 years	1658.60	30.25	1634.80	28.45	9123.60	21.95	9352.40	22.75
45–54 years	1472.20	26.85	1540.60	26.81	11004.80	26.48	9699.20	23.60
55–64 years	621.40	11.33	846.80	14.74	7520.40	18.10	8232.20	20.03
>64 years	134.80	2.46	160.00	2.78	3578.40	8.61	3645.60	8.87
Total	5483.60	100.00	5746.20	100.00	41559.20	100.00	41104.80	100.00
**Number of female employees, by age group**	**Mean (thousand)**	**%**	**Mean (thousand)**	**%**	**Mean (thousand)**	**%**	**Mean (thousand)**	**%**
25–34 years	1429.00	35.61	1450.60	32.69	6491.40	23.64	6930.80	24.83
35–44 years	1269.20	31.63	1354.80	30.53	6023.80	21.94	6278.80	22.49
45–54 years	965.60	24.06	1116.20	25.16	7983.60	29.07	7170.00	25.69
55–64 years	296.20	7.38	453.20	10.21	4801.20	17.48	5326.00	19.08
>64 years	53.00	1.32	62.20	1.40	2159.00	7.86	2207.20	7.91
Total	4013.00	100.00	4437.00	100.00	27459.00	100.00	27912.80	100.00

* Defined as 5 years before new criteria: 2006–2010 for Taiwan and 1997–2001 for Japan. † Defined as 5 years after new criteria: 2011–2015 for Taiwan and 2002–2006 for Japan.

Table S2. Mortality rate for cardiovascular and cerebrovascular diseases (CVDs) in Taiwan and Japan

Year	Taiwan				Japan			
	Crude rate*	Age-standardized*†	Overwork-related*	Rate Ratio (Overwork-related CVD rate ÷ Age-standardized CVD rate)	Crude rate*	Age-standardized*†	Overwork-related*	Rate Ratio (Overwork-related CVD rate ÷ Age-standardized CVD rate)
1990	144.21	210.69	-	-	212.11	144.44	-	-
1995	146.28	180.73	-	-	221.49	125.14	-	-
2000	143.43	149.98	-	-	230.77	109.04	0.20	0.18%
2005	146.98	125.70	-	-	248.01	96.32	0.79	0.82%
2010	151.01	106.51	0.57	0.53%	276.37	87.67	0.67	0.76%
2013	160.59	100.45	0.88	0.87%	306.07	86.07	0.69	0.80%

* Unit: per 100,000 population. † Based on World Health Organization's new World Standard Population. http://www.who.int/healthinfo/paper31.pdf. Accessed 7 June 2016

Source of data: Institute for Health Metrics and Evaluation (IHME) (2015). GBD Compare. WA, United States. http://vizhub.healthdata.org/gbd-compare. Accessed 7 June 2016
